# Relapse Rates With Paliperidone Palmitate in Adult Patients With Schizophrenia: Results for the 6-Month Formulation From an Open-label Extension Study Compared to Real-World Data for the 1-Month and 3-Month Formulations

**DOI:** 10.1093/ijnp/pyad067

**Published:** 2024-02-01

**Authors:** Ibrahim Turkoz, Mehmet Daskiran, Uzma Siddiqui, R Karl Knight, Karen L Johnston, Christoph U Correll

**Affiliations:** Janssen Research & Development, LLC, Titusville, NJ, USA; Janssen Research & Development, LLC, Titusville, NJ, USA; Janssen Research & Development, LLC, Titusville, NJ, USA; Janssen Research & Development, LLC, Titusville, NJ, USA; Janssen Scientific Affairs, LLC, Titusville, New Jersey, USA; Zucker Hillside Hospital, Department of Psychiatry, Glen Oaks, New York, USA; Donald and Barbara Zucker School of Medicine at Hofestra/Northwell, Department of Psychiatry and Molecular Medicine, Hempstead, New York, USA; Charité – Universitätsmedizin, Berlin, Department of Child and Adolescent Psychiatry, Germany

**Keywords:** Paliperidone palmitate 6-month, paliperidone palmitate 3-month, paliperidone palmitate 1-month, real-world data, relapse prevention, schizophrenia

## Abstract

**Background:**

The 3 paliperidone palmitate (PP) long-acting injectable antipsychotic formulations, PP 1-month (PP1M), PP 3-month (PP3M), and PP 6-month (PP6M), have shown to reduce the risk of relapse in schizophrenia. The current phase-4 study constructed external comparator arms (ECAs) using real-world data for PP3M and PP1M and compared relapse prevention rates with PP6M from an open-label extension (OLE) study in adult patients with schizophrenia.

**Methods:**

PP6M data were derived from a single-arm, 24-month, OLE study (NCT04072575), which included patients with schizophrenia who completed a 12-month randomized, double-blind, noninferiority, phase-3 study (NCT03345342) without relapse. Patients in the PP3M and PP1M ECAs were identified from the IBM^®^ MarketScan^®^ Multistate Medicaid Database based on similar eligibility criteria as the PP6M cohort.

**Results:**

A total of 178 patients were included in each cohort following propensity score matching. Most patients were men (>70%; mean age: 39–41 years). Time to relapse (primary analysis based on Kaplan-Meier estimates) was significantly delayed in the PP6M cohort (*P *< .001, log-rank test). The relapse rate was lower in the PP6M cohort (3.9%) vs PP3M (20.2%) and PP1M (29.8%) cohorts. Risk of relapse decreased significantly (*P *< .001) by 82% for PP6M vs PP3M (HR = 0.18 [95% CI = 0.08 to 0.40]), 89% for PP6M vs PP1M (HR = 0.11 [0.05 to 0.25]), and 35% for PP3M vs PP1M (HR = 0.65 [0.42 to 0.99]; *P* = .043). Sensitivity analysis confirmed findings from the primary analysis. Although the ECAs were matched to mimic the characteristics of the PP6M cohort, heterogeneity between the groups could exist due to factors including prior study participation, unmeasured confounders, variations in data capture and quality, and completeness of clinical information.

**Conclusions:**

In a clinical trial setting, PP6M significantly delayed time to relapse and demonstrated lower relapse rates compared with PP3M and PP1M treatments in real-world settings among adult patients with schizophrenia.

**Trial registration:**

ClinicalTrials.gov Identifier: NCT04072575; EudraCT number: 2018-004532-30

**Significance Statement**

Paliperidone palmitate (PP), an injectable antipsychotic medicine used to treat schizophrenia, decreases relapse risk (reappearance of clinically relevant symptoms). Relapse prevention rates of PP 6-month (PP6M), an injection administered once every 6 months, were compared with PP 3-month (PP3M; administered once every 3 months) and PP 1-month (PP1M; administered once every month). Data for PP6M were derived from a single-arm phase-3 clinical study in adults with schizophrenia. Data for PP3M and PP1M were obtained from the IBM^®^ MarketScan^®^ Multistate Medicaid Database, which collects real-life, routine medical and healthcare service use data for individuals enrolled in the US Medicaid program. The study showed that the longer-acting PP6M (in a clinical trial setting) substantially delayed the time to occurrence of relapse, with fewer patients experiencing relapse (3.9%) compared with the shorter-acting PP3M (20.2%) and PP1M (29.8%) in routine treatment settings among adults with schizophrenia. These findings could aid doctors’ treatment choices for maintaining effective therapy in patients with schizophrenia.

## INTRODUCTION

Relapses in schizophrenia increase the risk of treatment refractoriness, impose further stigmatization on patients, and have detrimental effects on the overall disease trajectory (Emsley et al., 2013). Nonadherence to schizophrenia medications resulting in treatment discontinuation is recognized as one of the primary risk factors for relapse (Robinson et al., 1999; Kemmler et al., 2005; Novick et al., 2010). Addressing nonadherence has proven to be a catalyst in developing safe and effective long-acting injectable (LAI) antipsychotics. Unlike their oral counterparts, LAI antipsychotics allow for less frequent dosing and facilitate adherence by providing reliable medication delivery, predictable pharmacokinetics, and transparent monitoring (Agid et al., 2010;Brissos et al., 2014;Correll et al., 2016). An updated systematic review and meta-analysis comparing LAIs with oral antipsychotics reported a significantly lower risk of hospitalization or relapse with LAI use (29 RCTs: risk ratio [RR] = 0.88 [95% CI .79 to .99], *P* = .033; 44 cohort studies: RR = 0.92 [0.88 to 0.98]; 28 pre-post studies: RR 0.44 [0.39 to 0.51], *P* < .0001) (Kishimoto et al., 2021).

Recent real-world studies have also reported reductions in hospital readmission rates and emergency room visits with LAI treatment versus oral antipsychotics, with evidence supporting a reduced healthcare burden of schizophrenia (Kim et al., 2020; Latorre et al., 2020). Furthermore, a systematic review of 19 clinical practice guidelines reported consistent agreement in terms of LAI treatment, with 13 guidelines recommending LAIs as maintenance therapy and 5 advocating LAI use in patients with first-episode schizophrenia (Correll et al., 2022). The use of LAIs has also been associated with improvements in tangible measures such as daily functioning and quality-of-life that are often valued by patients and encourage treatment adherence (Kaplan et al., 2013; Brissos et al., 2014; Correll et al., 2016; Blackwood et al., 2020).

Paliperidone palmitate 1-month (PP1M) and 3-month (PP3M) LAI formulations have shown meaningful symptomatic control, functional improvements, and reductions in hospitalization and relapse rates in several clinical studies (Gopal et al., 2010; Hough et al., 2010; Berwaerts et al., 2015; Savitz et al., 2016; Di Lorenzo et al., 2018). Evidence from real-world studies also suggests that PP1M and PP3M are more effective than oral antipsychotics in delaying time to relapse or treatment failure (Alphs et al., 2015; Emond et al., 2019a; Patel et al., 2020; Segarra et al., 2021).

The PP6M LAI formulation was developed to provide an extended dosing window of 6 months and is currently the first and only available twice-yearly treatment for adults living with schizophrenia (Milz et al., 2023). PP6M is approved for the maintenance treatment of schizophrenia in adult patients who have been clinically stabilized on PP1M or PP3M. In a 12-month, double-blind (DB), randomized, noninferiority phase 3 study, 2 sequential injections of PP6M demonstrated comparable efficacy to PP3M in preventing schizophrenia relapses without raising any new safety concerns (Najarian et al., 2022). Subsequently, the long-term efficacy of PP6M with low relapse rates (3.9%) over 2 years was reported in a phase 3, single-arm, open-label extension (OLE) study (Najarian et al., 2023). Investigating the comparative efficacy of PP6M versus PP3M and PP1M will provide insights into the clinical benefits of longer dosing intervals and fewer injections in preventing relapses in patients with schizophrenia.

The present phase 4 study was designed to leverage real-world data (RWD) in extending the interpretability of clinical study findings and generate comparative analysis to inform clinical decision-making. Thus, external comparator arms (ECAs) were created for PP1M and PP3M by utilizing RWD from the IBM^®^ MarketScan^®^ Multistate Medicaid Database (IBM MDCD) while PP6M data were obtained from the phase 3 OLE study. The primary objective was to compare the effectiveness of PP6M, PP3M, and PP1M formulations in delaying and preventing relapse among adult patients with schizophrenia.

## METHODS

### Study Design and Data Sources

This retrospective phase 4 study derived data for PP6M from a 24-month, single-arm, phase 3 OLE study (NCT04072575) (Najarian et al., 2023). The administrative claims data from the IBM MDCD were used to extract data for the PP1M and PP3M cohorts that served as the ECAs. The IBM MDCD contains healthcare claims data including coverage eligibility and service use for individuals enrolled in Medicaid or Medicaid-managed programs in the United States. The database contains records pertaining to enrollment (e.g., medical and prescription records of enrollment, including patient demographics and plan type), inpatient and outpatient services, long-term care (i.e., medical and pharmacy claims), and financial information for more than 10 million Medicaid beneficiaries from 10 states.

All cohorts included patients who received moderate or high doses of PP1M, PP3M, or PP6M injections. Patients were assigned to 1 of the 2 dose categories based on their PP injection dose at study entry (supplementary Table 1).

**Figure 1.**
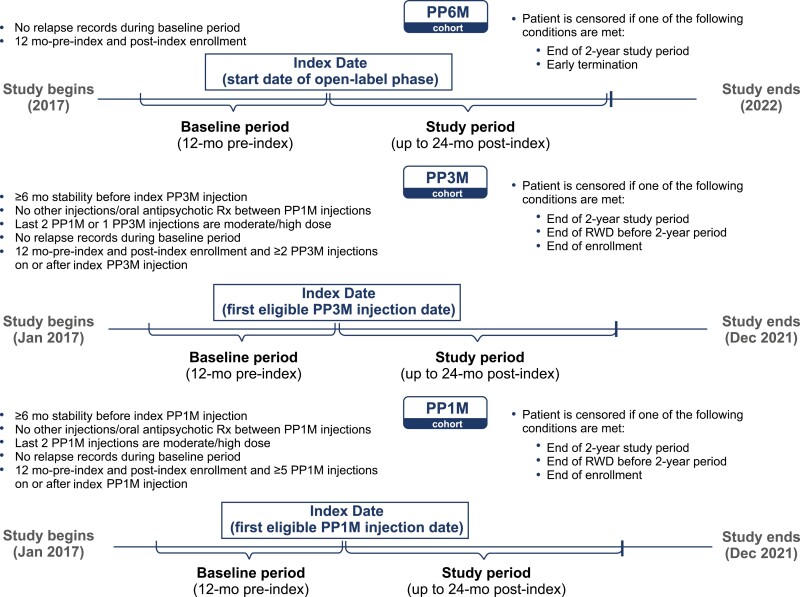
Study design. IBM MDCD, IBM MarketScan Multistate Medicaid Database; mo, month; PP1M, paliperidone palmitate 1-month formulation; PP3M, paliperidone palmitate 3-month formulation; PP6M, paliperidone palmitate 6-month formulation; RWD, real-world data; Rx, treatment.

The index date for the PP6M cohort was the start date (September 2019) of the OLE phase, and the end of the 12-month DB phase of the phase 3 study (NCT03345342) was considered the baseline period. The data collection period for the PP1M and PP3M cohorts was from January 1, 2017, until the end of available IBM MDCD data (last patient out December 2021) (Fig. 1). The date of the first eligible claim for PP1M or PP3M injection was deemed as the index date for the PP1M and PP3M cohorts. The baseline period for these cohorts was considered as the period between the index PP injection date and up to 12 months before this date. All patients had continuous medical and prescription coverage based on their insurance enrollment records during this period. The study follow-up period for all 3 cohorts was up to 24 months after the respective index dates. The IBM MDCD patients were required to have at least 1-year pre-index (baseline) and 1-year post-index continuous medical and drug coverage.

### Ethical Practices

The OLE study was conducted in accordance with the Declaration of Helsinki and applicable local laws and regulations; the study protocol was approved by an independent ethics committee or institutional review board. The present phase 4 study involved retrospective analyses of clinical study data and de-identified data from a claims database and is not considered human subject research. Therefore, an institutional review board approval or waiver was not required, and the study was exempted from the Health Insurance Portability and Accountability Act requirements.

### Patients

The PP6M cohort included patients who were transitioned from a randomized, DB, noninferiority phase study of PP6M (Najarian et al., 2022) and met the eligibility criteria for the OLE study (Najarian et al., 2023). The DB phase 3 study included patients aged 18 to 70 years with a diagnosis of schizophrenia (per the DSM-5) for ≥6 months before screening and a Positive and Negative Syndrome Scale total score of <70 points (no minimum cut-off) at screening. Patients were eligible for the OLE study if they had completed the 12-month DB phase without relapse, were willing to continue PP6M treatment, were deemed able to continue treatment at the same dose level (moderate or higher dose) as used during the DB phase of the noninferiority study at the time of screening for the OLE study, and were able to participate for the duration of the study.

Eligible patients in the PP3M and PP1M cohorts from the IBM MDCD were selected using similar inclusion and exclusion criteria as the PP6M cohort. The International Classification of Diseases procedure codes were identified and applied to create inclusion and/or exclusion criteria concepts. Eligible patients were ≥18 years of age (at index PP injection), received medium-/high-dose PP injections during the study period, were relapse-free during the 12-month baseline period, and had ≥1 year of continuous medical and prescription Medicaid insurance coverage before and after their index PP injection. Patients were included in the PP3M cohort if they received continuous treatment with PP1M or PP3M for ≥6 months before the index PP3M date. The last 2 doses of PP1M and first dose of PP3M were required to be the same and/or equivalent to the index PP3M dose. Similarly, patients in the PP1M cohort were included if they received continuous treatment with PP1M for ≥6 months before the index PP1M date. Subsequently, patients in the PP3M cohort received ≥2 injections, and those in the PP1M cohort received ≥5 injections after the corresponding index dates (supplementary Table 2). This criterion was incorporated to mimic the high completion rate observed in the OLE study (Najarian et al., 2023).

Patients who met any of the following criteria were excluded from the study: treatment with antipsychotics other than PP LAI within the 6-month pre-index date except for injectable risperidone; diagnosis of autism, dementia, manic episode, or bipolar disorder before the index date; the presence of any severe chronic conditions, such as diabetes with complications, cancer, heart conditions, coagulation disorders, or hematologic, pulmonary, thyroid, or renal diseases; body mass index >40 (morbidly obese patients); clozapine use within 2 months of the index date for PP injection. Patients enrolled with “medical insurance only” or indicating dual eligibility (both Medicare and Medicaid) were not included in the analysis.

### Propensity Score Matching (PSM)

PSM was used to enable adjusted comparisons between the cohorts and create a homogeneous subset of patients from the large real-world database, similar to patients from the phase 3 PP6M OLE study based on selected covariates. PSM was performed in the following order: PP3M-PP1M, PP6M-PP3M, and PP6M-PP1M. The propensity score model included covariates shown to affect both treatment assignment and outcomes as predictors.

For the PP3M-PP1M matching, all eligible PP3M and PP1M patients from the IBM MDCD database were included in the matching process. Propensity scores were calculated using the following factors: exact matching categories (index drug dose, age category, sex) and others (race, presence of baseline depressive disorder, Charlson Comorbidity Index score categories [0, 1 and >1], and Elixhauser Comorbidity Index score categories [0, 1, 2, 3 and >3]). The comorbidity scores were calculated using a medical coding algorithm (Quan et al., 2005). Patients were matched using a 1-to-1 nearest neighbor matching algorithm with a caliper of 0.2 SDs without replacement, such that the index drug dose, gender, and age category were an exact match. The success of matching was assessed using the standardized mean differences (SMD) for each covariate and SMDs before and after matching were compared. An SMD up to 0.20 was considered acceptable, and an SMD <0.10 was deemed a good match (Austin, 2014).

All PP6M patients were matched with the PP3M patients who were previously matched with eligible PP1M patients from the IBM MDCD database. Propensity scores were calculated using the relevant covariates chosen based on previous clinical findings and included PP dose, gender, and age categories. PP1M patients were then mapped to PP6M patients based on the previous PP6M-PP3M and PP3M-PP1M propensity scores.

### Relapse Analysis

The primary endpoint for the study was the time to first relapse. A relapse event was defined as the time to first occurrence of at least 1 or a combination of the following events during the study: psychiatric inpatient hospitalization for schizophrenia (involuntary or voluntary admission to a psychiatric hospital for decompensation of the patient’s schizophrenia symptoms); emergency room visits for worsening symptoms of schizophrenia not leading to a hospital admission (only PP6M cohort); self-injury or violent behavior resulting in a suicide attempt or injury to another person or significant property damage; suicidal or homicidal ideation. Emergency room visits that did not lead to a psychiatric hospitalization were not considered as relapse events in the PP3M and PP1M real-world cohorts because the visits could be for a variety of acute or surgical or medical reasons other than worsening of schizophrenia symptoms per se. For all 3 cohorts, if a patient relapsed, days to relapse were calculated based on the difference between the event date minus the index date +1. Patients who did not experience a relapse were censored.

### Statistical Analyses

No formal sample size determination was performed for this study. The primary analysis was based on a 1: 1: 1 greedy PSM approach. Kaplan-Meier survival curves were used to describe time to first relapse distributions in the 3 matched cohorts, and the reasons for relapses were summarized. Comparative analysis between the cohorts was based on log-rank test statistics. Hazard ratio (HR) and 95% confidence interval (CI) from the Cox proportional hazards models were computed to describe reduction in risk of relapses. The primary analysis was performed using the intent-to-treat analysis set, which included all patients who received PP6M during the phase 3 OLE study and matched PP3M and PP1M patients from IBM MDCD.

Robustness of the primary analysis was assessed using a sensitivity analysis based on a 1: 2: 2 matching ratio and a PSM approach, similar to the primary analysis. Model-based sensitivity analyses were also conducted to assess to what extent the primary analytical results become null using multi-bias E-value. The multi-bias E-value describes the minimum value that all of the sensitivity parameters for outcome misclassification, measurement error, and selection biases would have to take on for a calculated HR to be compatible with a truly null HR.

## RESULTS

### Propensity Score Matching

**Figure 2.**
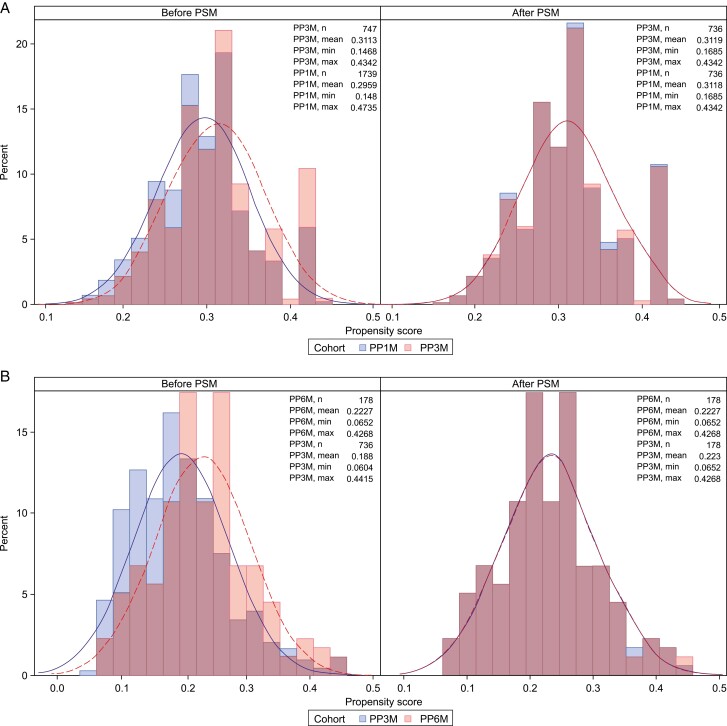
(A) PP3M and PP1M. (B) PP6M and PP3M propensity score distribution before and after PSM. PP1M, paliperidone palmitate 1-month formulation; PP3M, paliperidone palmitate 3-month formulation; PP6M, paliperidone palmitate 6-month formulation; PSM, propensity score matching.

The propensity score distribution was almost identical for the PP3M-PP1M, PP6M-PP3M, and PP6M-PP1M cohorts following PSM (Fig. 2). Using a 1: 1: 1 propensity score matching, 178 patients from each of the PP6M, PP3M, and PP1M cohorts who were the closest in propensity score values to their counterparts were selected for the primary analysis. Results indicated that after matching, the PP3M and PP1M cohorts derived from the RWD had comparable baseline patient characteristics to the PP6M cohort and therefore were used as ECAs in the study.

### Patient Disposition

A total of 178 patients from the DB phase 3 study who completed the 12-month DB phase were enrolled in the phase 3 OLE study as the PP6M cohort. All 178 patients enrolled in the OLE study were treated with at least 1 dose of PP6M and included in the primary analysis of the current study; of the 178 patients, 154 (86.5%) completed the study (supplementary Figure 1).

A total of 747 patients on PP3M and 1739 patients on PP1M from the IBM MDCD were included in the study, of which 736 patients from each cohort were successfully matched (PP3M-PP1M matching). After the subsequent PSM, 178 patients from each of the PP3M and PP1M cohorts were matched with the PP6M patients, serving as the ECAs for the primary analysis. In each of the PP3M and PP1M cohorts, a total of 128/178 patients (71.9%) completed the study. Patient withdrawal information for the PP3M and PP1M cohorts was not available, and patients could not complete the study due to either interruption in 2-year Medicaid eligibility or 2-year post-index data availability in the claims database.

### Demographics

Patient demographics before and after propensity matching were generally similar across the 3 cohorts (Table 1). The majority of SMDs post matching were <0.1, indicating well-balanced and optimally matched cohorts, with the largest SMD being 0.176, indicating an acceptable match. Patients included in the primary analysis had similar median ages of 40 (PP6M), 41 (PP3M), and 39 (PP1M) years. The majority of patients were men (>70%), and a high proportion of patients received high-PP index doses (>60%) across all 3 cohorts. The mean duration of total drug exposure was 682.1, 571.9, and 526.3 days for PP6M, PP3M, and PP1M, respectively.

**Table 1.** Demographic and baseline characteristics of patients in the PP6M, PP3M, and PP1M cohorts before and after PSM (ITT population)

Parameter	Before PSM			After PSM		
	PP6M n = 178	PP3M n = 736	PP1M n = 736	PP6M n = 178	PP3M n = 178	PP1M n = 178
**Sex, n (%)**						
Women	52 (29.2)	137 (18.6)	137 (18.6)	52 (29.2)	44 (24.7)	44 (24.7)
Men	126 (70.8)	599 (81.4)	599 (81.4)	126 (70.8)	134 (75.3)	134 (75.3)
SMD PP1M vs PP6M vs PP3M	0.250	0.000			0.101	0.000
SMD PP3M vs PP6M	0.250				0.101	
**Age category, n (%)**						
**18–25 y**	10 (5.6)	93 (12.6)	93 (12.6)	10 (5.6)	9 (5.1)	9 (5.1)
SMD PP1M vs PP6M vs PP3M	−0.246	0.000			0.025	0.000
SMD PP3M vs PP6M	−0.246				0.025	
**26–50 y**	135 (75.8)	527 (71.6)	527 (71.6)	135 (75.8)	138 (77.5)	138 (77.5)
SMD PP1M vs PP6M vs PP3M	0.096	0.000			−0.040	0.000
SMD PP3M vs PP6M	0.096				−0.040	
**≥50 y**	33 (18.5)	116 (15.8)	116 (15.8)	33 (15.8)	31 (17.4)	31 (17.4)
SMD PP1M vs PP6M vs PP3M	0.074	0.000			0.029	0.000
SMD PP3M vs PP6M	0.074				0.029	
**Index PP dose, n (%** **)**						
High	113 (63.5)	393 (53.4)	393 (53.4)	113 (63.5)	113 (63.5)	113 (63.5)
Moderate	65 (36.5)	343 (46.6)	343 (46.6)	65 (36.5)	65 (36.5)	65 (36.5)
SMD PP1M vs PP6M vs PP3M	0.206	0.000			0.000	0.000
SMD PP3M vs PP6M	0.206				0.000	
**Age**						
Mean (SD), y	40 (10.8)	37 (11.2)	37 (11.2)	40 (10.8)	41 (10.2)	39 (10.6)
SMD PP1M vs PP6M vs PP3M	0.301	0.000			0.152	0.176
SMD PP3M vs PP6M	0.300				−0.019	

Abbreviations: ITT, intent-to-treat; PP, paliperidone palmitate; PP1M, paliperidone palmitate 1-month formulation; PP3M, paliperidone palmitate 3-month formulation; PP6M, paliperidone palmitate 6-month formulation; PSM, propensity score matching; SMD, standardized mean differences.

### Primary Analysis for Relapse

**Figure 3.**
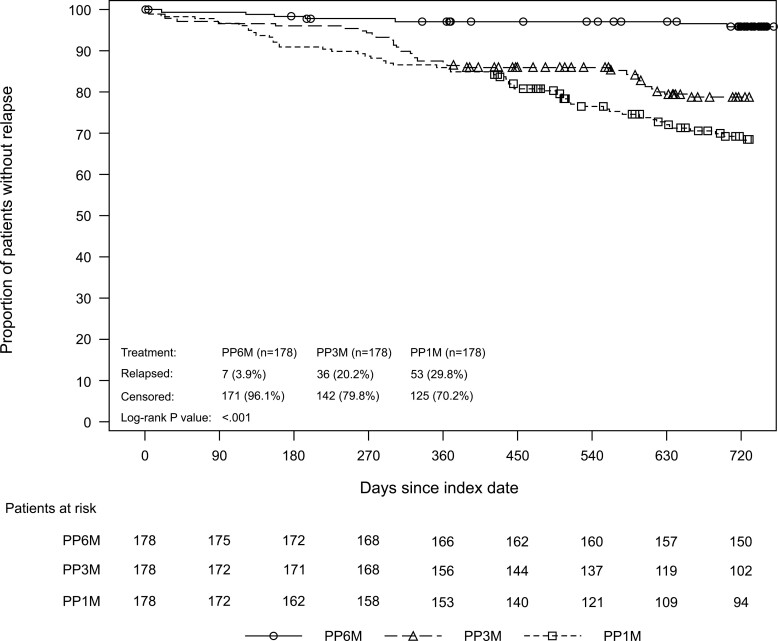
Kaplan-Meier estimate of time to first relapse (ITT population). ITT, intent-to-treat; PP1M, paliperidone palmitate 1-month formulation; PP3M, paliperidone palmitate 3-month formulation; PP6M, paliperidone palmitate 6-month formulation.

During the 2-year follow-up period, treatment with PP6M was associated with a significantly lower risk of relapse compared with PP3M and PP1M treatment. Patients in the PP6M cohort experienced a significant delay in time to relapse (*P* < .001), and the median time to relapse was not estimable for all 3 cohorts during the study period (Table 2; Figure 3). Overall, 3.9% (7/178) of patients in the PP6M cohort, 20.2% (36/178) in the PP3M cohort, and 29.8% (53/178) in the PP1M cohort experienced a relapse event. Risk of relapse decreased by 82% for PP6M vs PP3M (HR: 0.18 [95% CI: .08 to .40]; *P* < .001), 89% for PP6M vs PP1M (HR: 0.11 [95% CI: .05 to .25]; *P* < .001), and 35% for PP3M versus PP1M (HR: 0.65 [95% CI: .42 to .99]; *P *= .043). Psychiatric hospitalization was the most common reason for relapse in all 3 cohorts (PP6M: 2.2%; PP3M: 14.0%; PP1M: 25.8%) (Table 3).

**Table 2.** Risk of relapse analysis

	PP6M (n = 178)	PP3M (n = 178)	PP1M (n = 178)
**Primary analysis (ITT population)**			
Number assessed	178	178	178
Number censored (%)	171 (96.1)	142 (79.8)	125 (70.2)
Number of relapse (%)	7 (3.9)	36 (20.2)	53 (29.8)
Time to relapse (days)^a^			
25% percentile (95% CI)	NE	NE	NE
Median (95% CI)	NE	NE	NE
75% percentile (95% CI)	NE	NE	NE
Relative risk, HR (95 % CI)			
PP6M vs PP3M and PP1M	—	0.18 (0.08, 0.40)	0.11 (0.05, 0.25)
p-value	—	<0.001	<0.001
PP3M vs PP1M			0.65 (0.42, 0.99)
p-value			0.043
	**PP6M** **(n = 178)**	**PP3M** **(n = 356)**	**PP1M** **(n = 356)**
**Sensitivity analysis (sensitivity ITT population)**			
Number assessed	178	356	356
Number censored (%)	171 (96.1)	281 (78.9)	261 (73.3)
Number of relapse (%)	7 (3.9)	75 (21.1)	95 (26.7)
Relative risk, HR (95 % CI)			
PP6M vs PP3M and PP1M	—	0.17 (0.08, 0.36)	0.13 (0.06, 0.27)
*P* value	—	<0.001	<0.001
PP3M vs PP1M			0.76 (0.56, 1.03)
*P* value			0.074

Abbreviations: CI, confidence interval; HR, hazard ratio; ITT, intent-to-treat; NE, not estimable; PP1M, paliperidone palmitate 1-month formulation; PP3M, paliperidone palmitate 3-month formulation; PP6M, paliperidone palmitate 6-month formulation.

^a^Based on Kaplan-Meier estimates.

**Table 3.** Reasons for first relapse (ITT population)

	PP6M n = 178	PP3M n = 178	PP1M n = 178
Patients with relapse, n (%)	7 (3.9)	36 (20.2)	53 (29.8)
Reason for relapse^a^, n (%)			
Psychiatric hospitalization	4 (2.2)	25 (14.0)	46 (25.8)
Emergency room visit	2 (1.1)^b^	NA	NA
Suicidal ideation	2 (1.1)	6 (3.4)	13 (7.3)
Suicidal or homicidal ideation	2 (1.1)	0	1 (0.6)
Violent behavior resulting in suicide/injury or property damage	1 (0.6)	9 (5.1)	8 (4.5)
Homicidal ideation	1 (0.6)	2 (1.1)	1 (0.6)
Deliberate self-injury, violent behavior	1 (0.6)	1 (0.6)	1 (0.6)

Abbreviations: ITT, intent-to-treat; NA, not applicable; PP1M, paliperidone palmitate 1-month formulation; PP3M, paliperidone palmitate 3-month formulation; PP6M, paliperidone palmitate 6-month formulation.

^a^Reasons for relapse are not mutually exclusive; patients may have ≥1 reason for a given relapse.

^b^Two patients had emergency room visit due to worsening of schizophrenia symptoms and both resulted in hospitalization on the same day.

### Sensitivity Analysis for Relapse

PSM for the sensitivity analysis was performed using a 1: 2: 2 matching ratio and resulted in a final sample of 178 in the PP6M cohort and 356 patients each in the PP3M and PP1M cohorts. A total of 248/356 patients on PP3M (69.7%) and 247/356 patients on PP1M (69.4%) completed the study in the sensitivity analysis. Baseline patient characteristics for the sensitivity analyses before and after PSM were well-balanced across the 3 cohorts and comparable with the primary analysis (supplementary Table 3).

**Figure 4.**
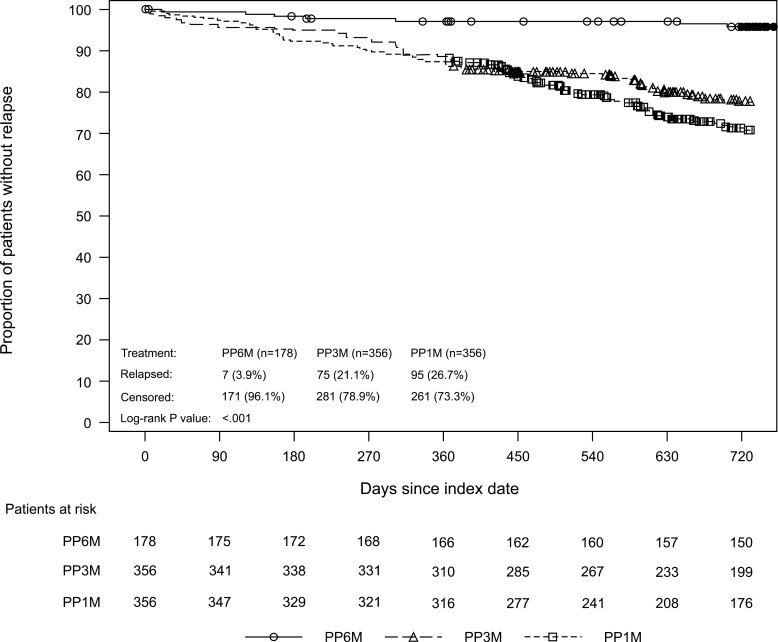
Kaplan-Meier estimate of time to first relapse (sensitivity ITT population). ITT, intent-to-treat; PP1M, paliperidone palmitate 1-month formulation; PP3M, paliperidone palmitate 3-month formulation; PP6M, paliperidone palmitate 6-month formulation.

Results from the sensitivity analysis revealed similar trends of relapse rates and time to relapse as observed in the primary analysis (Table 2; Figure 4). Treatment with PP6M was associated with a significantly lower relative risk of relapse compared with the PP3M (HR 0.17; *P* < .001) and PP1M cohorts (HR 0.13; *P* < .001). Relative risk between the PP3M and PP1M cohorts did not significantly differ (HR 0.76; *P *= .074). The rates of and reasons for first relapse were consistent with the primary intent-to-treat population, with the most common reason being psychiatric hospitalization (PP6M: 2.2%; PP3M: 16.6%; PP1M: 20.8%).

The multi-bias E-value was 3.05 for the calculated HR of 0.18 between PP6M vs PP3M. This E-value implies that all 3 parameters for each of the 3 assumed sources of bias (outcome misclassification, measurement error, and selection bias) would have to take a value of 3.05 for the observed HR between PP6M vs PP3M of 0.18 to be compatible with a true null effect. Similarly, a multi-bias E-value was 3.99 for the calculated HR of 0.11 between PP6M vs PP1M.

## DISCUSSION

This phase 4 study compared the effectiveness of PP6M with its shorter-acting counterparts, PP1M and PP3M, in delaying and preventing relapse among adult patients with schizophrenia. The present study is among the first few to construct ECAs (PP3M and PP1M cohorts) using RWD, enabling comparison of PP6M from an OLE study with PP3M and PP1M (Turkoz et al., 2023). The PSM yielded well-matched cohorts of PP6M, PP3M, and PP1M that were used for comparative analysis. Treatment with PP6M during the phase 3 OLE study significantly delayed time to relapse and was associated with a lower relapse rate (3.9%) compared with the matched real-world PP3M (20.2%) and PP1M (29.8%) arms. Supporting this finding, PP6M also demonstrated a clear advantage in terms of relapse prevention and showed the greatest magnitude of reduction in risk of relapses compared with PP3M and PP1M. The sensitivity analysis using less restrictive matching criteria included more patients in the ECA cohorts and yielded consistent results for PP6M, further substantiating the primary findings.

Over the years, injectable antipsychotic formulations with longer half-lives have been developed to provide extended medication exposure and potentially delay relapses even after treatment interruptions. Corroborating this hypothesis, a post hoc analysis reported longer relapse-free periods in patients who withdrew PP3M (13 months) compared with PP1M (6 months) or oral paliperidone extended-release formulation (2 months), with a significantly lower risk of relapse for PP3M vs PP1M (*P* < .001; Weiden et al., 2017). Pragmatic evidence from real-world studies has also shown the benefits of continuous medication exposure with LAI antipsychotics for improving medication adherence and lowering the rates of discontinuation and relapse in schizophrenia. In a retrospective analysis of health claims data from the IBM MDCD, patients in the PP3M vs PP1M cohort reported higher rates of adherence in terms of proportion of days covered (87% vs 78%; *P* < .0001) and corresponding lower relapse rates (10.5% vs 15.7%; Li et al., 2021). Similar analyses conducted with commercially insured patients who transitioned from PP1M to PP3M have reported comparable trends of improved adherence and treatment persistence and reduced healthcare resource utilization for schizophrenia and other comorbidities (Joshi et al., 2017; DerSarkissian et al., 2018; Emond et al., 2019b; Lin et al., 2021). Taken together, these observations are consistent with the current study, confirming the enduring benefits and efficacy of longer-acting LAIs such as PP6M in preventing relapses in patients with schizophrenia. PP6M offers an extended treatment window with twice-yearly dosing that could enhance adherence and help maintain clinical stability for improved long-term prognosis. Furthermore, PP6M may be beneficial for patients who are homeless, without caregiver support, or living in remote areas with limited proximity to healthcare providers (Sajatovic et al., 2013; Milz et al., 2023). Regardless of these circumstances, patients may still prefer PP6M LAI for the assurance of consistent medication delivery and extended protection with fewer yearly injections (Pietrini et al., 2019; Milz et al., 2023).

Pairing clinical trial data and high-quality RWD with adjustments for clinical covariates can broaden the understanding of treatment effects and allow rigorous evaluation of treatments (Yap et al., 2021). This study applied statistical and methodological approaches to generate ECAs for a PP6M OLE study using RWD for assessing clinical outcomes in patients with schizophrenia. External comparator designs are emerging as useful methods to generate supportive evidence and augment data from single-arm clinical studies while eliminating extensive resource requirements and high costs (Loiseau et al., 2022). However, causal inferences in external comparator studies with nonrandomized designs are prone to selection bias and confounding bias due to differences in baseline covariates. Therefore, a PSM approach was used to balance the baseline clinical characteristics of patients between the 3 cohorts and mitigate confounding and selection bias (Lu et al., 2022).

Leveraging RWD to construct ECAs allows access to large, clinically relevant populations of patients who may be underrepresented in clinical trials (Corrigan-Curay et al., 2018). The present study derived data from the Medicaid claims database, one of the largest payers for mental health services, and therefore is likely representative of the diverse population of patients with schizophrenia in the United States. The IBM MDCD collects standardized patient-level data on services and filled prescriptions allowing access to granular data on the key patient baseline, treatment, and demographic characteristics. Furthermore, patients in the real-world PP3M and PP1M cohorts were treated and followed up over the same time period as the patients in the PP6M OLE study (contemporaneous control), suggesting that the patients may have broadly comparable treatment histories, reflecting the ongoing standard of care (Carrigan et al., 2022).

Findings from this study should be considered in light of certain limitations inherent to RWD. In contrast to randomized controlled trials, ECAs derived from RWD cannot control for unmeasured or unknown confounders; PSM only allows adjustments for known confounders available in both the trial data and the claims database. Thus, factors such as substance abuse, presence of other psychiatric comorbidities, and need for other psychiatric comedications, which are common predictors of relapse, could not be matched. In some cases, relevant information recorded in the trial, such as laboratory values and mental health-related scales, may not be collected in the real-world dataset. Thus, multi-bias E-values were estimated to determine the potential impact of unmeasured and residual confounding on the findings. The large E-values suggest that unmeasured confounders are less likely to influence the observed results. For the IBM MDCD, although large, the selection of patients is limited to those insured under Medicaid from 10 states and may not be fully generalizable to patients who are not insured or are commercially insured. Additional research comparing PP6M with PP3M and PP1M using comprehensive data derived from patients enrolled in the US Medicaid program are warranted. Furthermore, patients with schizophrenia who have functional impairments are more likely to use healthcare resources and be included in the Medicaid pool, potentially leading to underdetection (Leucht et al., 2007). RWD reflects real-world patterns of care and varies in data quality and completeness. Inherent to any administrative claims database, the possibility of imprecise coding, incomplete clinical information, and delayed data entry could affect the validity of the current findings. Notably, claims databases reflect more of the “payer’s perspective” and less of the “physician” or “patient” perspectives, although relapse and hospitalization are outcomes that matter to all stakeholders. All clinical study outcomes could not be fully reproduced in the ECAs due to a lack of similar outcomes in the claims database. Thus, safety endpoints including adverse events were not assessed because comparable data for the PP3M and PP1M cohorts from the IBM MDCD were not available. Although important covariates that mimic the eligibility criteria and help minimize selection bias were considered, not all inclusion and exclusion criteria from the OLE study could be applied to patients in the IBM MDCD. Patients in the PP6M cohort showed a clear response to PP6M during the DB phase 3 study, which demonstrated relapse prevention with PP6M. The patients were adherent to PP6M under controlled clinical trial conditions during the OLE study. Thus, they may have had a higher likelihood of showing response than patients from the RWD-derived cohorts. It is also challenging to ascertain the treatment adherence among patients from the RWD ECAs because pharmacy claims may not necessarily indicate that patients received the medication and complied with the prescribing label. Additionally, the omission of emergency room visits in relapse assessment for the RWD cohorts could have impacted the estimation of relapse rates between PP6M and the RWD cohorts. These factors could introduce heterogeneity between the cohorts and affect the comparability of findings from the clinical study and RWD.

## CONCLUSIONS

In summary, well-matched ECAs of PP3M and PP1M were created using RWD from an administrative claims database to assess and compare outcomes with the PP6M cohort derived from a single-arm, phase 3 OLE study. Treatment with PP6M significantly delayed time-to-relapse and was associated with lower relapse rates and risk of relapse compared with the real-world derived PP3M and PP1M cohorts. These findings further support the use of PP6M as an effective maintenance therapy with the longest dosing window in the management of schizophrenia.

## Supplementary Material

pyad067_suppl_Supplementary_Tables_S1-S3_Figures_S1

## Statement of Interest

IT, MD, US, and RKK are employees of Janssen Research & Development, LLC (a Johnson & Johnson company) and may hold company stocks or stock options. KLJ is an employee of Janssen Scientific Affairs, LLC and stockholder of Johnson & Johnson. CUC has been a consultant and/or advisor to or has received honoraria from: AbbVie, Acadia, Alkermes, Allergan, Angelini, Aristo, Biogen, Boehringer-Ingelheim, Cardio Diagnostics, Cerevel, CNX Therapeutics, Compass Pathways, Darnitsa, Denovo, Gedeon Richter, Hikma, Holmusk, IntraCellular Therapies, Janssen/J&J, Karuna, LB Pharma, Lundbeck, MedAvante-ProPhase, MedInCell, Merck, Mindpax, Mitsubishi Tanabe Pharma, Mylan, Neurocrine, Neurelis, Newron, Noven, Novo Nordisk, Otsuka, Pharmabrain, PPD Biotech, Recordati, Relmada, Reviva, Rovi, Seqirus, SK Life Science, Sunovion, Sun Pharma, Supernus, Takeda, Teva, and Viatris. He provided expert testimony for Janssen and Otsuka. He served on a Data Safety Monitoring Board for Compass Pathways, Denovo, Lundbeck, Relmada, Reviva, Rovi, Sage, Supernus, Tolmar, and Teva. He has received grant support from Janssen and Takeda. He received royalties from UpToDate and is also a stock option holder of Cardio Diagnostics, Mindpax, LB Pharma, PsiloSterics, and Quantic.

## Data Sharing Statement

The data sharing policy of Janssen Pharmaceutical Companies of Johnson & Johnson is available at https://www.janssen.com/clinical-trials/transparency. As noted on this site, requests for access to the study data can be submitted through Yale Open Data Access [YODA] Project site at http://yoda.yale.edu.
