# Supplementary material for: Relapse Rates With Paliperidone Palmitate in Adult Patients With Schizophrenia: Results for the 6-Month Formulation From an Open-label Extension Study Compared to Real-World Data for the 1-Month and 3-Month Formulations
Source: Int J Neuropsychopharmacol. 2024 Feb 1;27(2):pyad067. doi: 10.1093/ijnp/pyad067 (PMC10873782; doi:10.1093/ijnp/pyad067)
Supplement: pyad067_suppl_Supplementary_Tables_S1-S3_Figures_S1 [file pyad067_suppl_supplementary_tables_s1-s3_figures_s1.docx]

**Supplementary Material**

**Supplementary Table S1: Dose conversions for PP1M, PP3M, and PP6M**

|  | **PP1M Dose** | | **PP3M Dose^a^** | | **PP6M Dose^b^** | |
| --- | --- | --- | --- | --- | --- | --- |
|  | **mg eq.** | **mg** | **mg eq.** | **mg** | **mg eq.** | **mg** |
|  | 25 mg eq. | 39 mg | - | - | - | - |
|  | 50 mg eq. | 78 mg | 175 mg eq. | 273 mg | - | - |
|  | 75 mg eq. | 117 mg | 263 mg eq. | 410 mg | - | - |
| **Moderate dose^c^** | 100 mg eq. | 156 mg | **350 mg eq.** | **546 mg** | **700 mg eq.** | **1092 mg** |
| **High dose^c^** | 150 mg eq. | 234 mg | **525 mg eq.** | **819 mg** | **1000 mg eq.** | **1560 mg** |
| ^a^PP3M dose = 3.5x the patient's previous PP1M dose; ^b^PP6M dose = 7x the patient's previous PP1M dose or approximately 2x the previous PP3M dose; ^c^Doses shown in bold represent the PP3M and PP6M doses evaluated during the double-blind, phase 3 study of PP6M.  **Abbreviations:** PP1M, paliperidone palmitate 1-month formulation, paliperidone palmitate 3-month formulation; PP6M, paliperidone palmitate 6-month formulation | | | | | | |

**Supplementary Table S2**: Attrition steps in determining eligible PP3M and PP1M patients in the IBM^®^ MarketScan^®^ Multistate Medicaid Database cohorts

| **Attrition step** | **Inclusion and exclusion criteria** | **Patients** | **Patients excluded, n (%)** |
| --- | --- | --- | --- |
| 1 | Inclusion: Patients in IBM MDCD (2008-2021) | 34825270 |  |
| 2 | Inclusion: Patients in IBM MDCD (2017-2021) | 21402280 | 13422990 (38.5) |
| 3 | Inclusion: Patients with PP3M and/or PP1M injection, 2017 forward | 34654 | 21367626 (99.8) |
| 4 | Inclusion: Patients with high/moderate dose PP3M and/or PP1M injection | 32071 | 2583 (7.5) |
| 5 | Inclusion: Adult patients (age ≥18) | 27512 | 4559 (14.2) |
| 6 | Inclusion: Patients with schizophrenia diagnosis prior to index PP injection | 24340 | 3172 (11.5) |
| 7 | Inclusion: Patients with ≥1 year pre- (baseline) and post-index PP injection enrollment | 14136 | 10204 (41.9) |
| 8 | Exclusion: Patients with relapse records during baseline period | 8020 | 6116 (43.3) |
| 9 | Exclusion: Patients with other pre-index mental condition diagnosis and clozapine use (2 months) | 6389 | 1631 (20.3) |
| 10 | Exclusion: Patients with severe chronic conditions | 4962 | 1427 (22.3) |
| 11 | Exclusion: Patients with baseline (6 months) Rx prescription known to prolong QC-interval or effect PP metabolism | 3862 | 1100 (22.2) |
| 12 | Exclusion: Patients with metabolic conditions (morbidly obese, underweight) and pregnancy/delivery | 3711 | 151 (3.9) |
| 13 | Inclusion: Adequately treated (5+ PP1M injections. With last 2 eq. to index dose). Must have ≥2 PP3M or 5 PP1M injections during study period | 2486 | 1225 (33.0) |
| - | **Eligible PP3M patients** | **747** |  |
| - | **Eligible PP1M patients** | **1739** |  |
| Note: Emergency room visit records are not included in relapse definition at exclusion step 8. **Abbreviations:** IBM MDCD, IBM^®^ MarketScan^®^ Multistate Medicaid Database; PP, paliperidone palmitate; PP1M, paliperidone palmitate 1-month formulation; PP3M, paliperidone palmitate 3-month formulation | | | |

**Supplementary Table S3: Demographic, baseline characteristics of patients in the PP6M, PP3M, and PP1M cohorts before and after PSM (Sensitivity ITT population)**

|  | **Before PSM** | | | | **After PSM** | | | |
| --- | --- | --- | --- | --- | --- | --- | --- | --- |
| **Parameter** | PP6M  n=178 | PP3M  n=736 | PP1M  n=736 | PP6M  n=178 | | PP3M  n=356 | PP1M  n=356 |  |
| **Sex, n (%)** |  | | | | | | | |
| Women | 52 (29.2) | 137 (18.6) | 137 (18.6) | 52 (29.2) | | 96 (27.0) | 96 (27.0) |  |
| Men | 126 (70.8) | 599 (81.4) | 599 (81.4) | 126 (70.8) | | 260 (73.0) | 260 (73.0) |  |
| SMD PP1M vs PP6M vs PP3M | 0.250 | 0.000 |  |  | | 0.050 | 0.000 |  |
| SMD PP3M vs PP6M | 0.250 |  |  |  | | 0.050 |  |  |
| **Age category, n (%)** |  | | | | | | | |
| **18–25 years** | 10 (5.6) | 93 (12.6) | 93 (12.6) | 10 (5.6) | | 18 (5.1) | 18 (5.1) |  |
| SMD PP1M vs PP6M vs PP3M | −0.246 | 0.000 |  |  | | 0.025 | 0.000 |  |
| SMD PP3M vs PP6M | −0.246 |  |  |  | | 0.025 |  |  |
| **26–50 years** | 135 (75.8) | 527 (71.6) | 527 (71.6) | 135 (75.8) | | 267 (75.0) | 267 (75.0) |  |
| SMD PP1M vs PP6M vs PP3M | 0.096 | 0.000 |  |  | | 0.020 | 0.000 |  |
| SMD PP3M vs PP6M | 0.096 |  |  |  | | 0.020 |  |  |
| **≥50 years** | 33 (18.5) | 116 (15.8) | 116 (15.8) | 33 (18.5) | | 71 (19.9) | 71 (19.9) |  |
| SMD PP1M vs PP6M vs PP3M | 0.074 | 0.000 |  |  | | −0.036 | 0.000 |  |
| SMD PP3M vs PP6M | 0.074 |  |  |  | | −0.036 |  |  |
| **Index PP dose, n (%)** |  | | | | | | | |
| High | 113 (63.5) | 393 (53.4) | 393 (53.4) | 113 (63.5) | | 228 (64.0) | 228 (64.0) |  |
| Moderate | 65 (36.5) | 343 (46.6) | 343 (46.6) | 65 (36.5) | | 128 (36.0) | 128 (36.0) |  |
| SMD PP1M vs PP6M vs PP3M | 0.206 | 0.000 |  |  | | −0.012 | 0.000 |  |
| SMD PP3M vs PP6M | 0.206 |  |  |  | | −0.012 |  |  |
| **Age** |  | | | | | | | |
| Mean (SD), years | 40 (10.8) | 37 (11.2) | 37 (11.2) | 40 (10.8) | | 41 (10.7) | 39 (11.0) |  |
| SMD PP1M vs PP6M vs PP3M | 0.301 | 0.000 |  |  | | 0.126 | 0.149 |  |
| SMD PP3M vs PP6M | 0.300 |  |  |  | | −0.023 |  |  |
| **Abbreviations:** ITT, intent-to-treat; PP, paliperidone palmitate; PP1M, paliperidone palmitate 1-month formulation; PP3M, paliperidone palmitate 3-month formulation; PP6M, paliperidone palmitate 6-month formulation; PSM, propensity score matching; SMD, standardized mean differences | | | | | | | | |

**Supplementary Figure S1: Patient selection and disposition flow diagram**


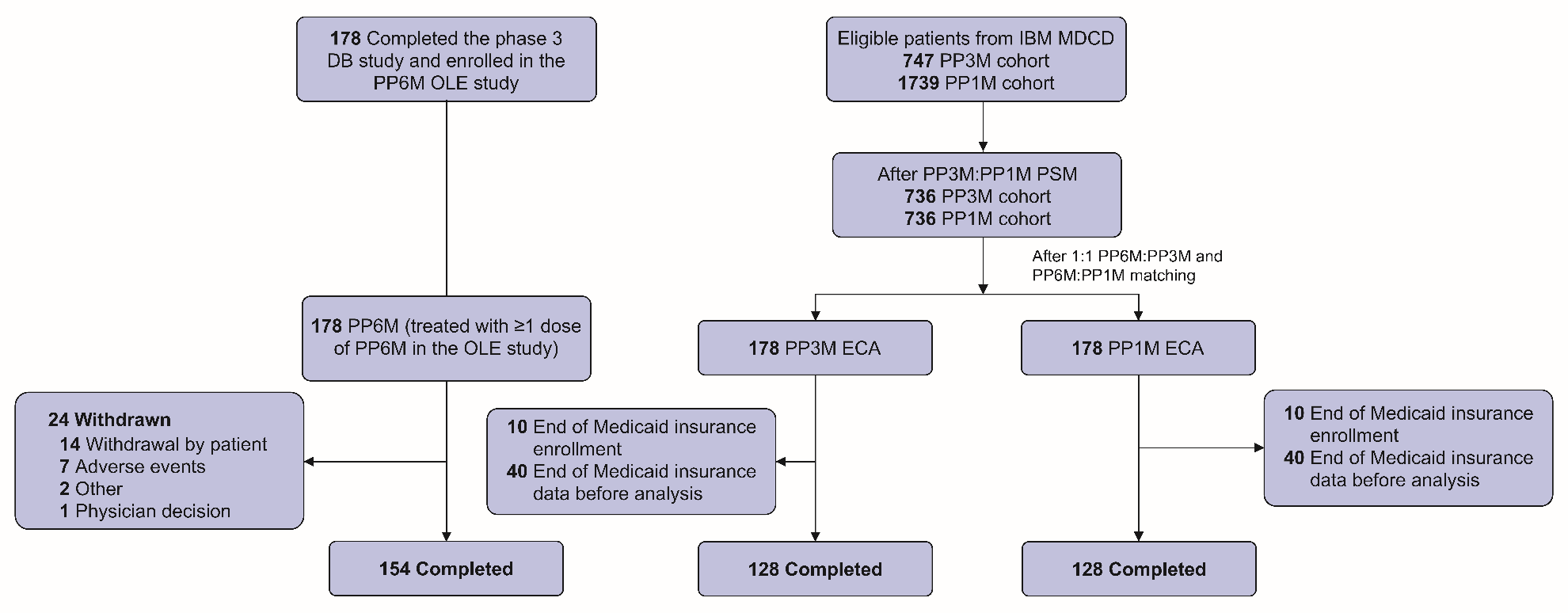


DB, double-blind; ECA, external comparator arm; IBM MDCD, IBM^®^ MarketScan^®^ Multistate Medicaid Database; OLE, open-label extension; PP1M, paliperidone palmitate 1-month formulation; PP3M, paliperidone palmitate 3-month formulation; PP6M, paliperidone palmitate 6-month formulation
